# Oncogenic nexus of cancerous inhibitor of protein phosphatase 2A (CIP2A): An oncoprotein with many hands

**DOI:** 10.18632/oncotarget.2127

**Published:** 2014-06-22

**Authors:** Pradip De, Jennifer Carlson, Brian Leyland-Jones, Nandini Dey

**Affiliations:** ^1^ Department of Molecular & Experimental Medicine, Avera Research Institute, Sioux Falls, SD; ^2^ Department of Internal Medicine, SSOM, University of South Dakota, Sioux Falls, SD

**Keywords:** CIP2A, PP2A, c-MYC, Cancers, Prognosis, Biomarkers

## Abstract

Oncoprotein CIP2A a Cancerous Inhibitor of PP2A forms an “oncogenic nexus” by virtue of its control on PP2A and MYC stabilization in cancer cells. The expression and prognostic function of CIP2A in different solid tumors including colorectal carcinoma, head & neck cancers, gastric cancers, lung carcinoma, cholangiocarcinoma, esophageal cancers, pancreatic carcinoma, brain cancers, breast carcinoma, bladder cancers, ovarian carcinoma, renal cell carcinomas, tongue cancers, cervical carcinoma, prostate cancers, and oral carcinoma as well as a number of hematological malignancies are just beginning to emerge. Herein, we reviewed the recent progress in our understanding of (1) how an “oncogenic nexus” of CIP2A participates in the tumorigenic transformation of cells and (2) how we can prospect/view the clinical relevance of CIP2A in the context of cancer therapy. The review will try to understand the role of CIP2A (a) as a biomarker in cancers and evaluate the prognostic value of CIP2A in different cancers (b) as a therapeutic target in cancers and (c) in drug response and developing chemo-resistance in cancers.

## INTRODUCTION

The unharnessed growth and metastasis of a tumor mass [1] is initiated either by a single and/or by a number of sequential multiple genetic triggers, the cumulative effects of which are known to manifest through certain discrete common growth promoting signaling pathways of cells. The entire course of growth and metastasis of cancer as a disease, is realized through simultaneous and/or successive deleterious genetic changes affecting a wide range of cellular functions either inside the cell itself (e.g. from DNA damage repair to antigen response) and / or outside the cell (e.g. from angiogenesis to the dissolution of matrix proteins). Thus the entire sequence of events of the growth and metastatic evolution of a tumor, although unique to each patient from the standpoint of its oncogenic events, course of growth, drug/radiation response and the development of resistance to drug/radiation is attributed to the long-lasting consequence of the genetic changes either in their oncogene(s), tumor suppressor(s) genes, or oncogenic transcription factors, which either singularly or collectively setup each patient's “oncogenic stage/background”.

**Figure 1:**
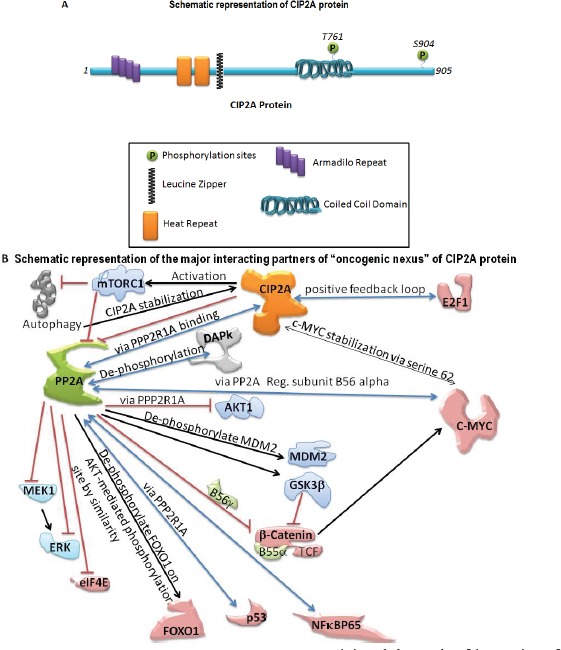
Schematic representation of the basic structure of CIP2A (A) and the mode of interaction of CIP2A with c-MYC and PP2A (B) The “oncogenic nexus” of CIP2A is primarily constituted of the interactive functional network between oncogenic transcription factors, tumor suppressors and different signaling components of the PI3K-mTOR pathway, the RAS-MEK-ERK pathway and the Wnt-beta-catenin pathway. Schematic representation of the major interacting partners of “oncogenic nexus” involving CIP2A protein has been compiled from *Puustinen, P., 2014; Perrotti, D. and Neviani, P., 2014; Li, Y., 2013; Khanna, A., 2013; Laine, A., 2013; Niemela, M., 2012; Westermarck, J. and Hahn, W. C. 2008; Junttila, M. R. and Westermarck, J. 2008; Junttila, M. R. et al., 2007.* Bi-directional blue arrows indicate interactions between two entities; uni-directional black arrows indicate a positive influence of one on the other entity; blocking red lines indicate a negative influence of one on the other entity. Transcription factors are color coded in pink. Signaling molecules of the RAS-MAPK-ERK pathway and the PI3K-AKT-mTOR pathway are coded in two different shades of blue.

Cancerous Inhibitor of PP2A, CIP2A (Recommended name: Protein CIP2A; Alternative name(s):p90 autoantigen) is a human onco-protein [2]. The basic structure of CIP2A is shown in Figure 1A. As an integral protein, CIP2A functions via protein binding through interactions with many proteins including PP2A, (a tumor suppressor), MYC, (a pleiotropic transcription factor; MYC proto-oncogene protein, a class E basic helix-loop-helix protein 39; Transcription factor p64), polo like kinase (PLK1), and NIMA (Never In Mitosis Gene A)-related kinase 2 (NEK2) protein. CIP2A [(Q8TCG1 (CIP2A_HUMAN) Reviewed, UniProtKB/Swiss-Prot Last modified May 14, 2014. Version 90)] has been reported to have binary interactions with MYC (MYC proto-oncogene protein; Entry: P01106) and PPP2R1A (serine/threonine-protein phosphatase 2A 65 kDa regulatory subunit A alpha isoform; Entry:P30153) (Binary interactions provide information about binary protein-protein interactions. The data presented in this section are a quality-filtered subset of binary interactions automatically derived from the IntAct database). CIP2A protein has been reported to have binary interactions wherein the interacting target(s) are FLT1 (Vascular endothelial growth factor receptor 1 Isoform Iso 2), MYC, and PPP2R1A (Source: neXtProtBETA).

*An “oncogenic nexus” of CIP2A refers to the interconnected regulatory network of CIP2A which is established either through direct (binary) interactions of CIP2A or indirectly through interactions of the CIP2A-PP2A duo with either multiple key cellular proteins/transcription factors* (onco-proteins like RAS, beta-catenin, c-SRC; tumor suppressors like PP2A, p53; transcription factors like MYC, E2F1, ETS1, ATF2, FLT1, CHK1) *or with components of key oncogenic pathways* (pathways like the PI3K-mTOR pathway, the RAS-MEK-ERK pathway, the Wnt-beta-catenin pathway) [3-10]. CIP2A by virtue of its functional interactions with a wide number of oncogenesis related proteins and transcription factors forms the major constituent of “oncogenic nexus”. *The central event of the “oncogenic nexus” constitutes the close and reciprocal functional interactions between CIP2A, c-MYC and PP2A which fine tunes the balance between the function of an oncogenic transcription factor, c-MYC and a tumor suppressor, PP2A* [11]. PP2A [2, 12, 13] constitutes one of the major tenets of the “oncogenic nexus” of CIP2A. CIP2A by itself does not constitute the “oncogenic nexus”; rather it forms the unique and irreplaceable component of the nexus. The major role of CIP2A in the “oncogenic nexus” is imparted to its control over another important component of the nexus, PP2A.

CIP2A controls oncogenic cellular signals by suppressing tumor suppressor PP2A [2, 12, 14]. Hence understanding the molecular structure, the function and the regulation of PP2A is crucial to envisage the “oncogenic nexus” of CIP2A [15]. CIP2A binds to PP2A and inhibits its phosphatase functions resulting in tumorogenic transformation of cells. PP2A has been identified as a protein involved in regulating c-MYC expression [11]. CIP2A stabilizes c-MYC towards oncogenic transformation. MYC is regulated by CIP2A via PP2A. Niemelä et al., have shown that depletion of certain PP2A subunits reverses CIP2A siRNA effects on both MYC and proliferation [16]. CIP2A interacts directly with c-MYC, inhibits PP2A activity toward c-MYC serine 62, and thereby prevents c-MYC proteolytic degradation. As serine 62 of MYC is an established PP2A target regulated by CIP2A, it appears that CIP2A functions towards MYC are similar to CIP2A's functions towards other PP2A target proteins. Thus CIP2A controls oncogenic transcription in tumor cells and the “oncogenic nexus” of CIP2A protein in human malignancies is executed through the stabilization of MYC protein involving PP2A. From the oncogenesis point of view, these changes converge on the oncogenic upregulation of the RAS-MAPK and the PI3K-mTOR pathways which help to transform cells [1, 15, 17]. PP2A and MYC dependent interactions of CIP2A which form the major components of the “oncogenic nexus” are shown in Figure 1B. The global effect of CIP2A on oncogenesis can be explained by CIP2A-mediated inhibition of PP2A and its consequent effects on a number of oncoproteins, tumor suppressors and transcription factors. Studies from multiple laboratories have so far demonstrated that CIP2A effects on regulating proliferation, migration, MYC and E2F1 are reversed by simultaneous PP2A inhibition.

There are also a number of PP2A-independent functions of CIP2A including (1) regulating the stability, localization and activity of PLK1 [18] (2) enhancing NEK2 kinase activity to facilitate centrosome separation [19] and (3) increasing self-renewal of neural progenitor cells [20]. Kim et al., reported that CIP2A depletion delayed mitotic progression, resulting in mitotic abnormalities independent of PP2A activity and CIP2A interacted directly with the polo-box domain of PLK1 during mitosis [18]. One of the studies that reported a PP1- and PP2A-independent function of CIP2A demonstrated the involvement of CIP2A in cell cycle progression through centrosome separation and mitotic spindle dynamics. Jeong et al., on the basis of their yeast two-hybrid and coimmunoprecipitation assays, demonstrated that NIMA (never in mitosis gene A)-related kinase 2 (NEK2) is a binding partner for CIP2A [19]. CIP2A exhibited dynamic changes in distribution, including the cytoplasm and centrosome, depending on the cell cycle stage in their study. Upon CIP2A depletion, centrosome separation and the mitotic spindle dynamics were impaired, resulting in the activation of spindle assembly checkpoint signaling and ultimately extension of the cell division time. This data can explain higher mitotic rates observed in many tumor cells upon an upregulation of CIP2A function as observed in several other studies. Their data implied that CIP2A strongly interacts with NEK2 during G2/M phase, thereby enhancing NEK2 kinase activity to facilitate centrosome separation in a PP1- and PP2A-independent manner [19]. In breast cancer cells, a positive feedback loop between CIP2A and E2F1 had been shown to define the cell-intrinsic senescence sensitivity [21]. Laine et al., showed that E2F1 overexpression, due to p53 or p21 inactivation, promotes expression of human oncoprotein CIP2A which in turn by inhibiting PP2A activity, increases stabilizing serine 364 phosphorylation of E2F1. Khanna et al., identified a novel functional link between DNA damage kinase CHK1 and regulation of the onco-protein CIP2A [22]. The clinical relevance of CIP2A as a CHK1 effecter protein was validated in several human cancer types including neuroblastoma where CIP2A was identified as an N-MYC-independent prognostic factor [23]. The role of JNK2/ATF2 in CIP2A regulation was first reported from Kallunki laboratory [24]. In the following years Zhao et al., also demonstrated that *Helicobacter pylori* enhanced CIP2A expression (mRNA and protein levels) and cell proliferation via JNK2/ATF2 signaling leading to malignant transformation in human gastric cancer cells [25]. The expression of CIP2A has been regulated both via several transcription factors as well as through downstream effectors of classical growth promoting steroid hormones dependent mitogenic pathways as reviewed in detail elsewhere [15]. Considering the close and overlapping functional ties between the CIP2A and PP2A, it is possible that many of CIP2A actions are directly or indirectly mediated via PP2A. Further experimental evidence is indeed required to provide convincing evidence for the PP2A-independent function of CIP2A which will implicate a better and clearer understanding of the complexity of CIP2A function and its functional relationship to PP2A. Thus future studies are warranted to prove whether these examples of CIP2A functions (“E2F1 regulation” and “as CHK1 target”) are functionally linked to PP2A or are independent of PP2A function.

The magnitude of the extent of involvement of CIP2A in the overall process of oncogenesis in different organ type can be envisaged by a recent article from Danish Cancer Society Research Center (Copenhagen, Denmark) who identified the regulatory circuit involving CIP2A and mTORC1 (as shown in Figure 1B) in tumor cells [26]. In their article Puustinen P et al., demonstrated that CIP2A associates with mTORC1. Through this interaction, CIP2A acts as an allosteric inhibitor of mTORC1-associated PP2A (PP2A negatively regulates mTORC1), thereby enhancing mTORC1-dependent growth signaling and inhibiting autophagy. Using ribonucleic acid interference screens for autophagy-regulating phosphatases in human breast cancer cells, they have identified that CIP2A acts as a key modulator of mTORC1 and autophagy. This regulatory circuit is reversed by ubiquitination and p62/SQSTM1-dependent autophagic degradation of CIP2A and subsequent inhibition of mTORC1 activity. An autophagic degradation of CIP2A upon mTORC1 inhibition leads to destabilization of c-MYC. In line with (a) CIP2A's reported ability to protect c-MYC against proteasome-mediated degradation [27] and (b) mTORC1's capability to integrate information regarding the availability of nutrients and energy to coordinate protein synthesis and autophagy [28-31], this evidence that CIP2A is functionally connected to the enhancement of mTOR function rationally strengthens the argument that CIP2A forms a dominant part of the oncogenic transformation in cells. In fact, Puustinen P et al.'s data not only characterize CIP2A as a distinct regulator of mTORC1 and reveals mTORC1-dependent control of CIP2A degradation as a mechanism that links mTORC1 activity with c-MYC stability to coordinate cellular metabolism, growth, and proliferation but also provides a strong evidence for the rationale that CIP2A as an oncopropein has the capability to control major aspects of a tumorogenic transformation of a cell. The complexity of the nexus is further amplified due to the involvement of mTORC, which negatively regulates PP2A activity [32-35] and studies by Li et al., detected increased PP2A activity in cancer cells exposed to rapamycin [35].

It is intriguing how the interactions of CIP2A (“oncogenic nexus”) with all different cellular components/signaling molecules function in complex co-ordinated ways to (1) enhance the activity of onco-proteins, (2) suppress the function of tumor suppressors, (3) stabilize pro-oncogenic transcription factors, (4) facilitate the function of other transcription factors and / or (5) control cell growth, protein synthesis and autophagy through growth factors, nutrients, energy sensors and mTORC1 which eventually signals towards oncogenic transformation of a cell. This review presents an “oncogenic nexus” of CIP2A involving PP2A and c-MYC in [2, 36] different cancers. The review describes the role of the PP2A-CIP2A oncogenic nexus in different organ type cancers and evaluates the clinical relevance of CIP2A “oncogenic nexus” in the context of therapeutic intervention.

### CIP2A in Cancers

CIP2A is overexpressed at a high frequency in a number of tumors and expression levels are independent markers for long-term outcomes in many of these tumors. There are reports of changes in the expression of CIP2A in both solid tumors and Myelodysplastic Syndromes. In solid tumors, CIP2A (mRNA or protein) has been shown to be amplified / overexpressed and these expression levels either correlated significantly with tumor stages or served as an independent prognostic marker for disease-free survival (DFS) and overall survival (OS). Interestingly the changes in the expression levels were also found to be tumor cell specific. CIP2A is rarely present (low levels) in non-transformed/non-malignant cells while in a typically transformed cell, CIP2A is present in abundance [37]. Supplementary Table 1 lists the reported of changes in CIP2A levels and its role in different cancers. Recently miR-375 has been reported to activate p21 and suppresses telomerase activity by coordinately regulating HPV E6/E7, E6AP, CIP2A and 14-3-3ζ in HPV-positive cancers [38]. In their study Jung et al demonstrated that miR-375-mediated repression of CIP2A, E6, E6AP and E7 occurs in HPV16-positive cells that simultaneously increases tumor suppressor p53, p21 and RB and causes cell cycle arrest.

### CIP2A in Colorectal Cancers

Wiegering et al., reported that CIP2A influences survival in colon cancer patients and is critical for maintaining MYC expression. CIP2A mRNA was amplified / overexpressed in colon tumor samples and CIP2A expression levels correlated significantly with tumor stage. They demonstrated that CIP2A serves as an independent prognostic marker for disease-free and overall survival [39]. Studies by Bockelman et al. reported that CIP2A overexpression is associated with c-MYC expression in colorectal cancer [40]. In another study Cristobal et al., reported that PP2A is frequently inactivated in patients with colorectal cancers indicating that PP2A represents a potential therapeutic target for this disease and its restoration using FTY720 shows promising therapeutic potential [41]. In their study a PP2A activator FTY720 impaired proliferation and clonogenic potential of cells, induced caspase-dependent apoptosis, affected AKT and ERK1/2 activation status and showed an additive effect with drugs used in standard chemotherapy (5-fluorouracil, SN-38 and oxaliplatin) in colorectal cancer patients.

### CIP2A in Lung Cancers

CIP2A is overexpressed in non-small cell lung cancer [42]. Although the mechanism of the involvement of CIP2A in lung cancer is not clearly understood, some recent studies have indicated the involvement of interleukin-10 (IL-10) in this event. The role of CIP2A in mediating interleukin-10 effect in the progression of human papillomavirus (HPV)-associated lung cancer has been recently studied [43]. The secretion of IL-10 in both malignant and immune cells is known to promote the progression of lung tumors hence it negatively impacts patient prognosis. The secretion of IL-10 by immune and malignant cells, as induced by the E6 protein of human papilloma virus type 16 or 18, contributed to tumor progression by upregulating CIP2A and MYC. HPV-infected lung cancer cells upregulated IL-10 at the transcriptional level (involving phosphorylation of cAMP responsive element binding protein 1 and CCAAT/enhancer binding protein β) which stimulates an autocrine loop relying on the IL-10 receptor (IL-10R) by binding to IL-10R expressed by immune cells and IL-10 in turn may imbalance T_H_1 vs. T_H_2 tumor-specific immune responses cumulatively favoring tumor progression. Above studies collectively indicate the involvement of CIP2A during malignant progression of lung tumors and argue in favor of the fact that IL-10 may aid in promoting tumor aggressiveness via upregulation of CIP2A transcription in lung adenocarcinoma [44].

### CIP2A in Osteosarcoma

CIP2A has been identified as a critical oncoprotein involved in cell proliferation and invasion, which could serve as a therapeutic target in osteosarcoma. Knockdown of CIP2A expression significantly reduced osteosarcoma cell proliferation and invasion, with decreased c-MYC expression and p-AKT expression. CIP2A depletion also facilitated apoptosis and inhibited MMP9 mRNA expression [45]. Considering the role of MMP9 and other members of MMP family in mediating invasion of tumor cells, inhibition of mRNA expression for MMP9 following depletion of CIP2A clearly indicated a direct effect of CIP2A in metastasis in this cancer.

### CIP2A in Esophageal and Gastric Cancers

CIP2A is overexpressed in esophageal squamous cell carcinoma [46] and increased CIP2A expression is a predictor of poor survival in esophageal cancer. Rantanen et al., demonstrated that there is a positive correlation between CIP2A and c-MYC expression (p = 0.018) in esophageal adenocarcinoma. Although according to adjusted Cox regression survival analysis CIP2A and c-MYC had no effect on survival, among patients with stage IVA-IVB cancer, there was a trend toward poor prognosis in CIP2A-positive patients. The expression of CIP2A and c-MYC were associated with one another, and in most cases of esophageal adenocarcinoma they were found to be co-overexpressed [47].

MYC-dependent regulation and prognostic role of CIP2A in gastric cancer has been reported by Khanna et al. [48]. In other study Li et al., reported that CIP2A is overexpressed in gastric cancer wherein CIP2A can serve as a biomarker and its depletion leads to an impaired clonogenicity of tumor cells [49]. CIP2A in tumor cells from malignant gastric tissues helped to maintain proliferation by preventing cell growth arrest, senescence, or differentiation and CIP2A expression is significantly (P < 0.001) discriminatory between normal and cancerous gastric tissue [49]. The relationship between specific bacterial infection and CIP2A-associated oncogenic changes has been demonstrated by Zhao et al., who proposed a model to explain the effect of CagA positive H. pylori infection on CIP2A in gastric epithelial cells. Their study demonstrated that the bacterial oncoprotein CagA protein [*Certain H. pylori strains have a 35-40 kb cag pathogenicity island (PAI), which is encoded by 27-33 genes; One of the constituents of the cag PAI is cagA that encodes a 120-140 kDa CagA protein*] was tyrosine-phosphorylated by non receptor tyrosine kinase, SRC kinase, and thereafter activated the MEK/ERK pathway to upregulate CIP2A expression in AGS cells. They also found that H. pylori infection-induced MYC stabilization was partially dependent on CIP2A expression, while CIP2A depletion caused increased MYC degradation [50].

### CIP2A in Pancreatic Cancers

Immuno-histochemical staining demonstrated that CIP2A expression correlated with poor tumor differentiation, TNM stage and lymph node metastasis in pancreatic ductal adenocarcinoma. Kaplan-Meier survival analysis of data from patients with CIP2A-positive expression showed lower overall survival rate than those with CIP2A-negative expression. Furthermore positive expression of CIP2A was strongly associated with loss of the epithelial marker E-cadherin and acquisition of the expression of the mesenchymal markers N-cadherin and vimentin suggesting that CIP2A might promote epithelial-mesenchymal transformation (EMT) and progression in pancreatic ductal adenocarcinoma, which together indicates that CIP2A may be a potential therapeutic target for patients with pancreatic ductal adenocarcinoma [51]. Recently Farrell et al., have reported that endogenous inhibitors of PP2A, SET (also known as I2PP2A) and CIP2A were overexpressed in human pancreatic cancer and contributed to decreased PP2A activity as well as overexpression and stabilization of the oncoprotein c-MYC [52]. Their results indicated that antagonizing SET and/or CIP2A may be an innovative approach for the treatment of human pancreatic cancer.

### CIP2A in Brain Cancers

The reports on the role of CIP2A in brain cancer are limited. Yi et al., reported the expression and biological role of CIP2A in human astrocytoma [53]. In their study to investigate the clinical significance and biological function of CIP2A in astrocytoma Yi et al., observed an overexpression of CIP2A which was positively correlated with advanced tumor grades. CIP2A depletion in the astrocytoma cell lines inhibited cell growth, reduced anchorage-independent cell growth and increased apoptosis. In addition CIP2A depletion increased caspase-3 cleavage and downregulated c-MYC, BCL2 and pAKT expression. Although this study indicates role of CIP2A as a clinically relevant oncoprotein and point out that CIP2A may be a promising therapeutic target of astrocytoma, further studies in this direction are required to draw a conclusion.

### CIP2A in Breast Cancers

CIP2A is associated with human breast cancer aggressiveness [54] and the overexpression of CIP2A has been shown to increase the proliferation of RAS/RAF mutated aggressive MDA-MB231 cell line [55]. Interestingly MDA-MB231, a RAS/RAF mutated cell line also is a MYC-dependent cell [56]. Yu et al., studied the expression and regulatory effects of CIP2A protein in breast cancer to report a correlation between CIP2A protein expression and the prognosis of breast cancer [57]. CIP2A signature revealed the MYC dependency of CIP2A-regulated phenotypes in the breast cancer. Niemelä et al., by studying the clinical relevance of the CIP2A-regulated transcriptome in breast cancer subtypes reported a high-confidence transcriptional signature that is regulated by CIP2A [16]. Bioinformatics pathway analyses of the CIP2A signature revealed that CIP2A regulates several MYC-dependent as well as MYC-independent gene programs [16]. CIP2A expression was also associated with *MYC* gene amplification (P<0.001). With regard to MYC, these results both validate CIP2A's role in regulating MYC-mediated gene expression and provide a plausible novel explanation for the high MYC activity in basal-like and HER2+ breast cancers. Laine et al., identified that the E2F1-CIP2A positive feedback loop is a key determinant of breast cancer cell sensitivity to senescence and growth arrest induction [21] the results of which may also facilitate stratification strategies for selection of patients to receive senescence-inducing cancer therapies. In a recent report, an important role for the E2F1-CIP2A feedback loop in causing senescence resistance in p53 compromised cancer cells has been demonstrated. It has been further proposed by Laine and Westermark that targeting of E2F1-CIP2A the feedback loop could provide a pro-senescence therapy that is effective in both p53- and RB-deficient cancer cells [58].

Results from studies in different organ-type cancers including breast cancer indicated that CIP2A, rather than independently/ exclusively accomplishing the tumorigenic effect in cells, forms an important component of the “oncogenic nexus” in concert with PP2A and c-MYC. Recently a report by Baldacchino et al., demonstrated that deregulation of PP2A is a common event in breast cancer and a particular subset of patients with suppressed PP2A activity are potentially eligible for treatment using therapies which target the PI3K/AKT/mTOR pathway such as phosphatase activators like FTY720 [59]. They reported that the cBioPortal for Cancer Genomics shows that 46.7% (245 cases out of 525 eligible cases) of all the subtypes of breast cancer patients either had a low expression, including deletions, of one of the PP2A complex components or a high expression, including amplification, of the inhibitory regulatory subunits (the criteria were generally mutually exclusive, except for PPP2CB and the PPP2R2A which can occur simultaneously). Furthermore 8.6% of the patients either had a high expression of CIP2A (KIAA1524) or a high expression of SET, an endogenous inhibitor of PP2A, which implied that the PP2A complex is sequestered in the cells. This in turn strengthens our argument that in a cell undergoing an oncogenic transformation, CIP2A activation may accompany a functional downregulation of PP2A either by mutation of its functional subunits or by high expression of its endogenous inhibitor, SET.

### CIP2A in Bladder Cancers

Huang et al., reported that CIP2A protein is specifically expressed in human bladder tumors. CIP2A is preferentially expressed in high-grade and high-stage TCC tumors, which are high-risk and invasive tumors. Their studies supported the role of CIP2A in bladder cancer progression and indicated the usefulness of CIP2A for the surveillance of recurrence or progression of human bladder cancer [60]. In another study, CIP2A was also reported as a predictor of survival and a novel therapeutic target in bladder urothelial cell carcinoma [61].

### CIP2A in Ovarian Cancers

CIP2A is overexpressed in human ovarian cancer and its expression has been found to regulate cell proliferation and apoptosis. Fang et al., reported that 65.79% of all the tumors in their study showed CIP2A overexpression including serous carcinomas (68.48%), endometrioid carcinomas (63.64%), mucinous carcinomas (52.17%) and clear cell carcinomas (100%). CIP2A overexpression positively correlated with advanced FIGO stage and tumor grade. CIP2A depletion in ovarian cancer cell lines inhibited proliferation, blocked cell cycle progression, increased paclitaxel-induced apoptosis, downregulated cyclin D1, c-MYC, p-RB, BCL2 and pAKT expression validating the role of CIP2A as a clinically relevant oncoprotein as well as establishing CIP2A as a promising therapeutic target of ovarian cancer [62]. Böckelman et al., reported that CIP2A protein expression is a novel marker of reduced survival in serous ovarian cancer patients [63].

### CIP2A in Other solid Cancers

CIP2A is overexpressed in human cholangiocarcinoma tissues, which correlated with poor prognosis and the expression of CIP2A protein was an independent prognostic factor for cholangiocarcinoma patients [64]. Expression of CIP2A in renal cell carcinomas correlated with tumor invasion, metastasis and patients' survival [65]. High CIP2A immuno-reactivity was an independent prognostic indicator in early-stage tongue cancer [66]. CIP2A was overexpressed in cervical cancer [67] and its expression was upregulated by human papillomavirus 16 E7 oncoprotein [68]. CIP2A expression was also increased in prostate cancer [69]. CIP2A expression and localization in oral carcinoma and dysplasia has been reported in different studies [70, 71]. Repression of CIP2A coding sequence was reported as the mechanism by which tumor suppressor miR-375 regulated MYC expression [72] in oral cancers. Furthermore CIP2A gene polymorphisms and hepatocellular carcinoma susceptibility has been reported [73]. CIP2A is highly expressed in hepatocellular carcinoma and its expression predicts poor prognosis [74, 75]. Recent studies by Wei et al., have demonstrated that miR-218 regulated the biological process of melanoma development by targeting the 3'-UTR of the oncogenes CIP2A and BMI1 and thus observed that CIP2A and BMI1 knockdown phenocopies miR-218 overexpression [76]. Their studies show that miR-218 plays a pivotal role in the development of the disease and by targeting CIP2A and BMI1, miR-218 regulates the proliferation, migration and invasion of the melanoma cell lines A375 and SK-MEL-2, explaining miR-218's pivotal role in melanoma development.

### CIP2A in Myeloid Cancers

CIP2A is over-expressed in acute myeloid leukaemia and associated with HL60 cells proliferation and differentiation [77]. Overexpression of CIP2A in bone marrow cells from a group of patients with a high-risk of myelodysplastic syndromes (MDS) has been reported by Li et al., who demonstrated that CIP2A plays an important role in the progression of myelodysplastic syndromes [78]. IHC analysis revealed that a patient having refractory anemia with excess blasts exhibited significant expression of CIP2A in bone marrow hematopoietic cells, while all patients with refractory cytopenia with unilineage or multilineage dysplasia and the control group were negative. CIP2A was mainly expressed by the MPO-positive myeloid series of cells and partly by the CD34-positive cells in association with the expression of phosphorylated c-MYC (p-c-MYC) protein and the cell cycle-related proteins Ki-67 and geminin. The percentage of phospho-c-MYC-positive cells in the bone marrow of CIP2A-positive MDS cases was significantly higher than that in CIP2A-negative MDS cases (P < 0.01). The expression levels of mRNA for CIP2A and PP2A exhibited positive correlation in MDS/control bone marrow. The data indicated that up-regulated expression of CIP2A might play a role in the proliferation of blasts in the MDS bone marrow and in disease progression in at least some cases. Increased expression of CIP2A has been also reported in aggressive subtypes of B-cell lymphoma by Lilja et al. [79]. CIP2A levels at diagnosis of chronic myeloid leukemia are known as a critical determinant of the disease progression [80]. CIP2A is also overexpressed in acute myeloid leukaemia and associated with HL60 cells proliferation and differentiation [77]. CIP2A was not only associated with the proliferation of the tumor cells or the progression of the disease, it was also found to be associated with the chromosomal translocation in these cancers. Coenen et al., identified CIP2A (KIAA1524) as a novel MLL translocation partner in acute myeloid leukemia [81]. Odero MD and colleagues had showed that PP2A inactivation is a recurrent event in acute myeloid leukemia (AML) and that overexpression of SET (I2PP2A) is a poor prognostic factor in this disease [82-84]. The fact that restoration of tumor suppressor activity by PP2A-activating drugs has anti-leukemic effects in both KIT-positive and KIT-negative AML cells suggests that salvaging PP2A function could represent an innovative therapeutic target in AML.

### A cross-cancer alteration summary for CIP2A (Gene Name: KIAA1524)

**Figure 2:**
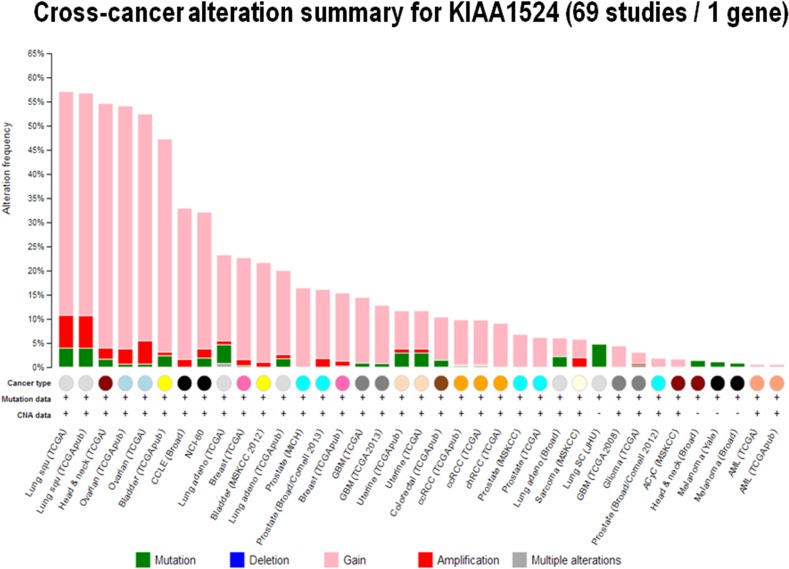
Changes in CIP2A in different cancers: Cross-cancer alteration summary for KIAA1524 (69 studies / 1 gene): The graph was generated using c-BioPortal Tumor types (tumor data sets) are chosen in accordance with the publication guidelines (last updated on January 17th, 2014) of TCGA (tcga@mail.nih.gov). have prioritized “Mutation and CNA” data type (selected KIAA1524: GAIN, AMP, MUT,). We acknowledge the cBioPortal for Cancer Genomics site (http://cbioportal.org) which provides a Web resource for exploring, visualizing, and analyzing multi-dimensional cancer genomics data. The portal reduces molecular profiling data from cancer tissues and cell lines into readily understandable genetic, epigenetic, gene expression and proteomic events (Gao et al., 2013, Integrative Analysis of Complex Cancer Genomics and Clinical Profiles Using the cBioPortal, Sci. Signal., 2 April, Vol. 6, Issue 269, p. pl1[DOI: 10.1126/scisignal.2004088]). We acknowledge works of Cerami et al. The cBio Cancer Genomics Portal: An Open Platform for Exploring Multi-dimensional Cancer Genomics Data [85, 86]. *Cancer Discovery*. May 2012 2; 401. PMID: 22588877 and Gao et al. Integrative analysis of complex cancer genomics and clinical profiles using the cBioPortal. *Sci. Signal.* 6, pl1 (2013). PMID: 23550210. We acknowledge the TCGA Research Network for generating TCGA datasets.

**Figure 3:**
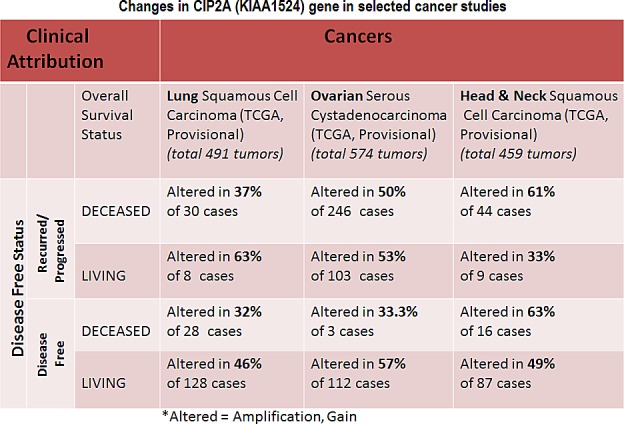
Chart showing changes in CIP2A (KIAA1524) gene in selected cancer studies including lung squamous cell carcinoma, ovarian serous cystadenocarcinoma and Head and Neck squamous cell carcinoma in the context of clinical attributions: A custom case set was build for the number of matching cases of lung squamous cell carcinoma using c-BioPortal (TCGA, Provisional; Lung Squamous Cell Carcinoma data set containing 489 samples; raw data at the NCI). Following Genomic Profiles were selected: (1) mutations, (2) putative copy-number alteration (CNA) from GISTIC, (3) mRNA expression Z-scores (RNA Seq V2 RSEM) with Z-score thresholds ± 2.0 and (4) protein/phospho-protein level (RPPA) with Z-score thresholds ± 2.0. (Total 230 samples). The custom case set was build for (A) Disease Free Status (this group represented the data either from the patients whose disease recurred / progressed or the patients who were disease free) and (B) Overall Survival Status(this group represented the data either from the deceased patients or from the patients who were alive).A custom case set was build for the number of matching cases of ovarian serous cystadenocarcinoma (TCGA, Provisional; TCGA Ovarian Serous Cystadenocarcinoma, containing 575 samples; raw data at the NCI.) using c-BioPortal. Following Genomic Profiles were selected: (1) mutations, (2) putative copy-number alteration (CNA) from GISTIC, (3) mRNA expression Z-scores (RNA Seq V2 RSEM) with Z-score thresholds ± 2.0 and (4) protein/phospho-protein level (RPPA) with Z-score thresholds ± 2.0. (Total 488 samples). The custom case set was build for (A) Disease Free Status (this group represented the data either from the patients whose disease recurred / progressed or the patients who were disease free) and (B) Overall Survival Status(this group represented the data either from the deceased patients or from the patients who were alive).A custom case set was build for the number of matching cases of Head and Neck squamous cell carcinoma (TCGA, Provisional; TCGA Head and Neck Squamous Cell Carcinoma, containing 426 samples; raw data at the NCI.) Following Genomic Profiles were selected: (1) mutations, (2) putative copy-number alteration (CNA) from GISTIC, (3) mRNA expression Z-scores (RNA Seq V2 RSEM) with Z-score thresholds ± 2.0 and (4) protein/phospho-protein level (RPPA) with Z-score thresholds ± 2.0 (Total 166 samples). The custom case set was build for (A) Disease Free Status (this group represented the data either from the patients whose disease recurred / progressed or the patients who were disease free)and (B) Overall Survival Status(this group represented the data either from the deceased patients or from the patients who were alive). We acknowledge the cBioPortal for Cancer Genomics site (http://cbioportal.org) which provides a Web resource for exploring, visualizing, and analyzing multi-dimensional cancer genomics data. The portal reduces molecular profiling data from cancer tissues and cell lines into readily understandable genetic, epigenetic, gene expression and proteomic events (Gao et al., 2013, Integrative Analysis of Complex Cancer Genomics and Clinical Profiles Using the cBioPortal, Sci. Signal., 2 April, Vol. 6, Issue 269, p. pl1[*DOI: 10.1126/scisignal.2004088*]). We acknowledge works of Cerami et al. The cBio Cancer Genomics Portal: An Open Platform for Exploring Multidimensional Cancer Genomics Data [85, 86]. *Cancer Discovery*. May 2012 2; 401. PMID: 22588877 and Gao et al. Integrative analysis of complex cancer genomics and clinical profiles using the cBioPortal. *Sci. Signal.* 6, pl1 (2013). PMID: 23550210. We acknowledge the TCGA Research Network for generating TCGA datasets.

**Figure 4:**
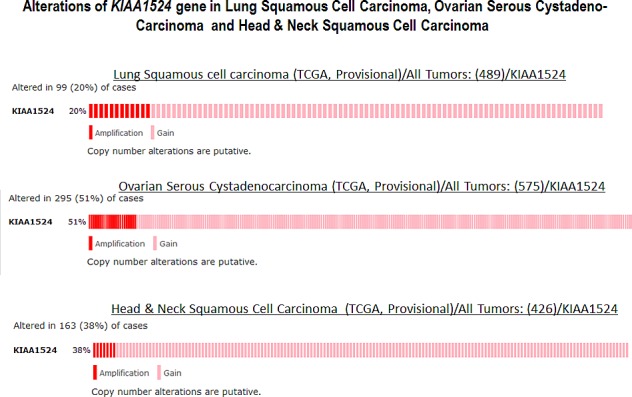
Alterations (Amplification and Gain) of KIAA1524 gene in Lung Squamous Cell Carcinoma (TCGA, Provisional), Ovarian Serous Cystadenocarcinoma (TCGA, Provisional) and Head & Neck Squamous Cell Carcinoma (TCGA, Provisional). Data was obtained using c-bioPortal. Unaltered cases were removed We acknowledge the cBioPortal for Cancer Genomics site (http://cbioportal.org) which provides a Web resource for exploring, visualizing, and analyzing multi-dimensional cancer genomics data. The portal reduces molecular profiling data from cancer tissues and cell lines into readily understandable genetic, epigenetic, gene expression and proteomic events (Gao et al., 2013, Integrative Analysis of Complex Cancer Genomics and Clinical Profiles Using the cBioPortal, Sci. Signal., 2 April, Vol. 6, Issue 269, p. pl1[*DOI: 10.1126/scisignal.2004088*]). We acknowledge works of Cerami et al. The cBio Cancer Genomics Portal: An Open Platform for Exploring Multidimensional Cancer Genomics Data [85, 86]. *Cancer Discovery*. May 2012 2; 401. PMID: 22588877 and Gao et al. Integrative analysis of complex cancer genomics and clinical profiles using the cBioPortal. *Sci. Signal.* 6,pl1 (2013). PMID: 23550210. We acknowledge the TCGA Research Network for generating TCGA datasets.

Although CIP2A has been shown to be overexpressed in a number of solid as well as myeloid cancers, it is evident that there are only a handful of reports regarding the involvement of CIP2A in each of the organ type cancers. We have performed a cross-cancer alteration summary for KIAA1524 (69 studies / 1 gene) using c-Bioportal (Figure 2). Data mining was carried out using cBioPortal for Cancer Genomics, a data portal (cBioPortal for Cancer Genomics [85]), available at http://www.cbioportal.org to measure the incidence of conditions that are associated with the alterations in KIAA1524 gene, as per the criteria mentioned in the legends of respective figures (Fig. 2-4). The database query was based on deregulation (mutant, copy number alterations and altered expression) of the KIAA1524 gene. Tumor types (tumor data sets) are chosen in accordance with the publication guidelines (last updated on January 17^th^, 2014) of TCGA (tcga@mail.nih.gov). We have prioritized “Mutation and CNA” data type. (We acknowledge the cBioPortal for Cancer Genomics site (http://cbioportal.org) which provides a Web resource for exploring, visualizing, and analyzing multidimensional cancer genomics data. We also acknowledge the TCGA Research Network for generating TCGA datasets). Since the portal reduces molecular profiling data from cancer tissues and cell lines into readily understandable genetic, epigenetic, gene expression and proteomic events [86], we have generated a graph representing a cross-cancer alteration (mutations and putative copy-number alterations from GISTIC) summary for KIAA1524.

Data show that out of different organ type cancers harboring genetic changes in CIP2A the most predominant alterations (mutations and putative copy-number alterations from GISTIC) occurred in lung squamous cell carcinoma in which the Gene Set / Pathway was altered in more than 50% of all cases (Lung Squamous Cell Carcinoma, TCGA, Nature 2012/Tumors with sequencing and aCGH data: (178)/User-defined List/1gene). Out of this the mutation occurred in less than 4% cases while the amplification occurred in more than 6% cases, while more than 40% cases showed “Gain”. In an individual cancer type the ratio of mutation to amplification varied from 1 (as in cervical cancer; data not shown) to mutation > amplification as in melanoma, bladder, uterine (data not shown) to amplification > mutation as in ovarian cancer and head & neck cancers. Certain cancers harbored only mutations as in bladder cancers, stomach cancers, lung adenocarcinoma, colorectal cancers, GBM and pancreatic cancers (data not shown). Prostate adenocarcinoma and sarcoma (data not shown) exhibited only amplification (Figure 2). When (1) mutations, (2) putative copy-number alteration (CNA) from GISTIC, (3) mRNA expression Z-scores with Z-score thresholds ± 2.0 and (4) protein/phospho-protein level (RPPA) with Z-score thresholds ± 2.0 were selected, the alterations were found higher than that observed for only mutations and putative copy-number alteration (CNA) from GISTIC in most of the cancers. For example, KIAA1524 gene is altered (AMP, GAIN) in ~20% of all 491 lung squamous cell carcinoma cases (TCGA, Provisional; TCGA Lung squamous cell carcinoma, containing 491 samples; raw data at the NCI), altered in ~52% of all 574 ovarian serous cystadenocarcinoma cases (TCGA, Provisional; TCGA ovarian serous cystadenocarcinoma, containing 574 samples; raw data at the NCI), and altered in ~36% of all 459 Head and Neck squamous cell carcinoma cases (TCGA, Provisional; TCGA Head and Neck squamous cell carcinoma, containing 459 samples; raw data at the NCI). We have selected three organ type cancers with higher percentage of changes in CIP2A including lung squamous cell carcinoma, ovarian serous cystadenocarcinoma and Head and Neck squamous cell carcinoma in the context of clinical attributions and tabulated the changes in CIP2A (KIAA1524) gene. The custom case sets for individual cancer were build for (A) Disease Free Status (this group represented the data either from the patients whose disease recurred / progressed or the patients who were disease free) and (B) Overall Survival Status (this group represented the data either from the deceased patients or from the patients who were alive) (Figure 3). The table shows that in Head and Neck carcinomas, the alterations in KIAA1524 were higher in the patients whose disease recurred/progressed as compared to disease free patients. However this pattern was found opposite in ovarian serous cystadenocarcinoma and lung carcinomas. When each of the recurred/progressed and disease free groups were broken down into deceased and living in Head and Neck cancer, the percentage of the change in the gene was found higher in the deceased group that the living group, a pattern opposite to that has been found in the lung squamous cell carcinoma patients. It will need a greater and in-depth study involving a higher number of cases to establish any association between the change in the KIAA1524 gene and the clinical attributes towards determing the prognostic value of KIAA1524. Considering the contribution of CIP2A in the process of tumorigenesis in different cancers, we have also determined the alterations (Amplification and Gain) of KIAA1524 gene in Lung Squamous Cell Carcinoma (TCGA, Provisional), Ovarian Serous Cystadenocarcinoma (TCGA, Provisional), and Head & Neck Squamous Cell Carcinoma (TCGA, Provisional) using c-bioPortal (Figure 4). Ovarian Serous Cystadenocarcinoma exhibited more than 50% of cases (out of 575) where an alteration (either gain or amplification) was recorded. We have not observed a clear seperation of the line representing patients showing no alteration from the line representing patients showing the alteration in the Overall Survival curve (OS; Kaplan-Meier estimate).

Additionally dominant types of alterations in KIAA1524 gene which were observed across different organ-type cancers were in the category/level of “gain” under data type of copy number alterations (CNA). Most of these “gain(s)” were observed in cervical squamous cell carcinoma and endocervical adenocarcinoma (TCGA, Provisional), where the gain was observed in 55.6% (20 cases out of 36 total cases) cases (c-BioPortal data; not presented due to limitation of permission). The other cancers which exhibited a similar order of frequency of the alteration (gain) in KIAA1524 were lung squamous cell carcinoma, head and neck squamous cell carcinoma, ovarian serous cystadenocarcinoma, and bladder urothelial carcinoma. It appears from the results from different laboratories who studied the tumorigenic involvement of CIP2A, PP2A and c-MYC in different organ-type cancers, that CIP2A forms an important component of the “oncogenic nexus” in concert with PP2A and c-MYC in achieving the tumorigenic effect in cells. Recently Baldacchino et al., reported that the cBioPortal for Cancer Genomics shows that PP2A is deregulated in 59.6% of basal type of breast tumors [59]. We have also observed that many of these cancer types like ovarian serous cystadenocarcinoma, bladder urothelial carcinoma and head and neck squamous cell carcinoma with higher frequencies of gain in KIAA1524 gene also have high frequencies of alterations in c-MYC oncogene (both gain and amplification) (data not shown). Considering the role of CIP2A protein in the stabilization of c-MYC protein, it will be worthwhile to look for an additional relationship between these two oncoproteins in coordinating an oncogenic transformation in cells. However it is beyond the scope of our current review to critically evaluate the nature of relationship between these two genes and their respective proteins.

It appears from the data that there is an upregulation of the genetic message for KIAA1524 across different organ type cancers specially those exhibiting a “gain” around 50%. Considering the role of protein product of KIAA1524 gene in cells, it is possible that this event is link to oncogenic transformations. Two facts are in favor of this argument. First the product of KIAA1524 gene CIP2A is a proto-oncoprotein and second, CIP2A is overexpressed at high frequency (40-80%) in most of the human cancer types (*as discussed in this review*). But the strongest support for this conclusion comes from the systematic analysis by Khanna et al., towards the contribution of potential gene regulatory mechanisms for high CIP2A expression in cancer [87]. Searching for the mechanisms of induction of CIP2A expression in cancer, they identified proximal −27 to −107 promoter region responsible for MEK-dependent stimulation of CIP2A expression (two functional ETS1 sites on the proximal CIP2A promoter) and reported that ETS1 acts as the transcription factor mediating stimulation of CIP2A expression through the EGFR-MEK pathway. CIP2A mRNA expression was sensitive to inhibition of EGFR activity as well as inhibition or activation of the MEK-ERK pathway. Khanna et al., in their bioinformatics analysis of overexpression of CIP2A and components of the EGFR-MEK1/2-ETS1 pathway from two different genome wide leukemia studies have identified M6 subtype of acute myeloid leukemia as a cancer type in which CIP2A and representative genes of each level of the pathway (EGFR, MEK2 and ETS1) were significantly upregulated. The result of the study demonstrate that the EGFR-MEK1/2-ETS1 pathway is a critical positive regulator of CIP2A expression revealing a potential link between deregulated EGFR-MEK1/2-ETS1 pathway signaling and CIP2A-dependent tumor growth [87]. In contrast to the role of ETS1 alone in the transcriptional control of CIP2A as reported by Khanna et al., in prostate and gastric carcinomas, the later reports by Pallai et al., showed that additional factors also regulate CIP2A expression in a cell-type specific manner [88]. Pallai et al., have characterized the proximal promoter region of the human CIP2A gene in cervical, endometrial and liver carcinoma cells to demonstrate that the 5' flanking minimal proximal promoter of the CIP2A gene consists of putative binding sites for ETS1 and ELK1 in forward and reverse orientations. Pallai et al.,demonstrated that in cervical, endometrial and liver carcinoma cell lines, the binding of both ETS1 and ELK1 to the proximal CIP2A promoter is absolutely required for CIP2A expression. ETS1 and ELK1 binding was found essential for the basal expression of CIP2A in several urogenital cancer cell lines. This observation is complementary to our observation that bladder urothelial carcinoma exhibited a high order of frequency in the alteration (gain) in KIAA1524 (Fig. 2). However the patho-physiological relationship between the “gain” in KIAA1524 gene and the transcriptional expression of CIP2A protein remains unresolved.

### CIP2A as Biomarkers in Cancers: Prognostic Value

The “oncogenic nexus” of CIP2A has provided some advantages in the choice/use of certain drugs. It has been reported that CHK1 targeting reactivated PP2A tumor suppressor activity in cancer cells via CIP2A [23]. Studies from Khanna et al., suggest that because the CHK1-CIP2A-PP2A pathway is driven by DNA-PK activity, functioning regardless of p53 or ATM/ATR status, which may (1) explain how CHK1 inhibitors mediate single-agent anticancer efficacy and (2) define CIP2A-PP2A status in cancer cells as a pharmacodynamic marker for the response to CHK1-targeted therapy [23].

CIP2A expression may be a potential biomarker for chemotherapeutic sensitivity and prognosis in breast cancer [57]. Interestingly Liu et al., demonstrated that auto-antibodies against p90/CIP2A may be useful serum biomarker for early stage breast cancer screening and immuno-diagnosis [89].

CIP2A is highly expressed in hepatocellular carcinoma and its expression predicted poor prognosis in this cancer [74, 75]. CIP2A gene polymorphisms and hepatocellular carcinoma susceptibility has been reported [73]. Bortezomib (a proteosome inhibitor used in clinics on myeloma patients) congeners induced apoptosis in hepatocellular carcinoma cells via CIP2A inhibition [90]. The inhibition of CIP2A has been shown to determine the effect of bortezomib on apoptosis and PP2A-dependent AKT inactivation in hepatocellular carcinoma indicating that CIP2A may be a biomarker for predicting clinical response of bortezomib in hepatocellular carcinoma treatment [91].

CIP2A regulates bortezomib-induced apoptosis in leukemia cells [92]. Cancerous inhibitor of protein phosphatase 2A was expressed in leukemic blasts from bone marrow samples. Ectopic expression of CIP2A upregulated pAKT and protected HL-60 cells from bortezomib-induced apoptosis, whereas silencing CIP2A overcame the resistance to bortezomib-induced apoptosis in MOLT3 and K562 cells. Bortezomib also exerted *in vivo* antitumor activity in HL-60 xenografted tumors and induced cell death in some primary leukemic cells indicating a major role in mediating bortezomib-induced apoptosis in leukemia cells.

A prognostic role for CIP2A expression has been reported in serous ovarian cancer [63]. CIP2A protein expression is a novel marker of reduced survival in serous ovarian cancer patients [63]. Their study concluded that CIP2A could be used to predict biological behavior in the group of patients with otherwise favorable prognosis. The result suggested that CIP2A characterized (sub-classified) the aggressive type of the disease even within subgroups with initially favorable prognosis. The association of CIP2A expression with survival evaluated by Kaplan-Meier method demonstrated that CIP2A immuno-positivity is a marker of reduced overall survival. Positive CIP2A expression was more frequently observed with high grade, advanced stage, aberrant p53 immuno-reactivity, high proliferation index, and aneuploidy of tumor cells [63]. Even in subgroups of patients with favorable clinical factors, CIP2A expression was strongly associated with reduced survival.

Huang et al., identified and evaluated CIP2A (both mRNA or protein) as a novel reliable and sensitive biomarker for diagnostics (early detection) in cervical cancer [93]).Their studies indicated that CIP2A (mRNA/protein) was specifically expressed (1) in cervical cancer tissues (different cancer stages), but not in non-cancer or adjacent non-tumor cervical tissue and (2) in cervical cell lines, but not in normal epithelial cell lines. The data strongly indicated that only CIP2A (but not PP2A or c-MYC) is a reliable biomarker for detection of cervical cancer and furthermore there was no strong correlation of CIP2A expression with HPV subtype, age, ethnical background, or other patient characteristics.

Studies undertaken by Huang et al., to test the expression of CIP2A for bladder cancer diagnostics demonstrated CIP2A as a novel bladder cancer biomarker [94]. In their study CIP2A protein was found specifically expressed in bladder tumor tissue at different cancer stages like most of other solid tumors, and not in adjacent non-tumor bladder tissue. CIP2A protein was also abundantly expressed in bladder cancer cell lines while it was not expressed in non-neoplastic epithelial cell lines.

The p90/CIP2A has been referred to as a fetal onco-protein in lung cancer [95]. Expression data for CIP2A in lung cancer also supported the working hypothesis that auto-antibody production in cancer may be directly linked to aberrant expression of proteins involved in tumorigenesis pathways. Peng et al., identified an immune response to p90/CIP2A in lung cancer [95]. In order to address the possibility whether or not the p90/CIP2A might be a tumor-associated antigen (TAA) and a useful biomarker in lung cancer, they used the full-length recombinant p90/CIP2A protein as the antigen in an enzyme-linked immunoassay (ELISA) and Western blotting was performed for the detection of auto-antibodies in 105 sera from patients. Of the 72 lung cancer tissue specimens examined, increased expression of p90/CIP2A was observed in 61 (84.7%) specimens, which was significantly higher than in normal lung tissues (14.3%, 9/63). Data indicated that tested together with antibodies against other well-validated TAAs such as p53, p62/IMP2, auto-antibody to p90/CIP2A might provide a potential novel marker for lung cancer detection. In other studies, overexpressed CIP2A correlated with poor prognosis in non-small cell lung cancers [42]. CIP2A expression in non-small cell lung cancers correlated with TNM stage, while survivin expression correlated with TNM stage and lymph node metastasis. Kaplan-Meier survival analysis showed that the overall survival times in patients expressing either CIP2A or survivin protein in non-small cell lung cancers were shorter. The expression of CIP2A protein was an independent prognostic factor for non-small cell lung cancers patients (COX regression analysis). Therefore CIP2A expression in non-small cell lung cancers patients may be an useful biomarker of malignancy [96].

The prognostic significance of CIP2A has been reported in several other malignancies including cancers of skin, stomach, colon/rectum and pancreas. CIP2A has proved its prognostic significance in cutaneous malignant melanoma (CMM). In their study Shi et al., demonstrated that CIP2A immuno-staining level was correlated with Breslow thickness, Clark's Level and lymphovascular invasion and high-CIP2A expression was associated with poor survival for patients, while *in vitro* downregulation of CIP2A attenuated metastasis of CMM cells [97]. CIP2A is a predictor of poor prognosis in colon cancer [98]. Peng et al., investigated the clinical significance of the expression of CIP2A in human colorectal cancer and examined the association of CIP2A expression with clinico-pathology and prognosis to show that up-regulated CIP2A expression is closely related to clinico-pathology of colorectal cancer. Their study indicated that CIP2A may be used as a potential predictive marker of metastasis, prognosis and therapeutic target in colorectal cancer [99]. MYC-dependent regulation and prognostic role of CIP2A is reported in gastric cancer [48]. Multivariate analysis as reported by Wang et al., showed that CIP2A expression is associated with altered expression of epithelial-mesenchymal transition (EMT) markers and is an independent prognostic factor for pancreatic ductal adenocarcinoma patients [51].

### CIP2A as Therapeutic Target

Based on the experimental evidence that CIP2A has a clinical relevance in the progression of the disease it has been regarded that CIP2A inhibitors have potential for use in the treatment in cancers. So far CIP2A has been targeted in a limited number of cancers using (1) natural compounds, (2) fusogenic-oligoarginine peptide-mediated delivery of siRNAs for gene silencing and (3) erlotinib derivatives.

Recently CIP2A has been targeted by a natural compound celastrol in non-small-cell lung cancer. Liu et al., demonstrated that celastrol binds CIP2A and enhances the CIP2A-CHIP interaction, leading to ubiquitination/degradation of CIP2A. Inhibition of CIP2A by celastrol inhibited the growth of lung cancer cells *in vitro* and *in vivo*. Their study also showed that celastrol potentiates cisplatin's efficacy by suppressing the CIP2A-AKT pathway indicating that CIP2A inhibitors may represent novel therapeutics for this cancer [100].

CIP2A has been targeted using fusogenic-oligoarginine peptide-mediated delivery of siRNAs in oral cancer cells. Cantini et al., demonstrated that a chimeric peptide consisting of a fusogenic sequence, in combination with cell-penetrating residues, can be used to effectively deliver siRNAs into oral cancer cells and induce the silencing of its target gene, potentially offering a novel therapeutic strategy involving CIP2A in combating oral cancer [101].

Studies have been undertaken in search of a lead compound for the development of CIP2A inhibitor for cancer therapies. In one of these studies a natural compound, ethoxysanguinarine (ESG), has been found to be effectively downregulating CIP2A protein and its downstream signaling molecules, c-MYC and pAKT, and ESG has been found to induce protein phosphatase 2A (PP2A) activity [102]. In their study Liu et al., also demonstrated that ESG inhibited proliferation and induced apoptosis of lung cancer cells and enhanced the effects of cisplatin on malignant cells.

Shiau et al., reported development of erlotinib derivatives which closely resemble erlotinib structurally but are devoid of tyrosine-kinase inhibitory activity as CIP2A inhibitors [103]. TD-19 (erlotinib derivative) had a higher efficacy than erlotinib on growth inhibition and apoptosis in erlotinib-resistant non-small cell lung cancer H460 and H322 cell lines through the CIP2A-PP2A-AKT pathway. Mechanistically their data also show that overexpression of CIP2A upregulated pAKT and protected H460 cells from TD-19-induced apoptosis [104]. CIP2A represents a major factor through which erlotinib derivatives induce apoptosis in hepatocellular carcinoma cells. The di-substituted erlotinib derivatives were tested for their ability to inhibit CIP2A and mediate cancer-cell proliferation. A correlation was observed between cell death, CIP2A and AKT inhibition by these derivatives. Inhibition of CIP2A determined erlotinib-induced apoptosis in hepatocellular carcinoma [105]. Erlotinib derivatives as CIP2A-ablating agents independent of EGFR activity have also been developed [106].

### Role of CIP2A in Drug response and Developing Chemo-Resistance in Cancers

The central role of CIP2A in the “oncogenic nexus” allows CIP2A to mediate the effects (cancer cell phenotypes) of many anti-cancer drugs. These effects not only confirm the pivotal role of CIP2A in the “oncogenic nexus”, but also helps to understand the mechanism of action of CIP2A in (1) the cellular transformation (oncogenic) and the progression of cancer cells (e.g. EMT), (2) mediating different drug responses (as drug target), (3) mediating drug-induced resistance and (4) determining the prognosis of a disease.

In line with its role as an oncogenic protein in the transformation of cells and the progression of a disease, CIP2A has been shown to play a role in mediating a number of drug effects in cancer. In hepatocellular carcinoma cells, Chen et al., reported that CIP2A mediates the effect of bortezomib on pAKT and apoptosis [91]. Following their observation that CIP2A is involved in mediating the apoptotic effect of bortezomib in hepatocellular carcinoma, Yu et al., also reported a proteasome-independent mechanism by which bortezomib induces autophagy in hepatocellular carcinoma through the CIP2A-PP2A-AKT-4EBP1 pathway [107]. The *in vivo* study showed that bortezomib downregulated CIP2A and upregulated PP2A activity in Huh-7 tumors. Ectopic expression of CIP2A decreased AKT-related PP2A activity whereas silencing CIP2A increased this activity, indicating that CIP2A negatively regulates AKT-related PP2A activity in hepatocellular carcinoma cells [91]. CIP2A-mediated AKT activation also plays a role in bortezomib-induced apoptosis in head and neck squamous cell carcinoma [108]. Bortezomib sensitizes hepatocellular carcinoma cells to CS-1008, an anti-human death receptor 5 antibody, through the inhibition of CIP2A [109]. CIP2A has been shown to be expressed in leukemic blasts from bone marrow samples. Liu et al., reported that bortezomib exerted *in vivo* antitumor activity in HL-60 xenografted tumors and induced cell death in some primary leukemic cells. Their study revealed that CIP2A plays a major role in mediating bortezomib-induced apoptosis in leukemia cells [92]. Inhibition of CIP2A is reported to be one of the mechanism by which bortezomib-mediated enhancement of radiation-induced apoptosis occurs in different solid tumors [110]. Treatment with bortezomib plus radiation downregulated CIP2A in a dose-dependent manner. Knockdown of CIP2A enhanced radiation-induced apoptosis in cancer cells, and ectopic expression of CIP2A in cancer cells abolished radiation-induced apoptosis. Thus bortezomib sensitized solid tumor cells to radiation through the inhibition of CIP2A. It suggests bortezomib may sensitize solid tumor cells to radiation through the inhibition of CIP2A. Tseng et al., also demonstrated that CIP2A mediates bortezomib-induced apoptosis also in TNBC [111] which is in line with their previous reports that CIP2A mediated effects of bortezomib on pAKT and apoptosis in hepatocellular carcinoma.

Tseng et al., has shown that CIP2A may be a novel target in breast cancer cells [112]. CIP2A expression is readily detectable in tumor samples from TNBC patients. Liu et al., showed that bortezomib inhibited CIP2A in association with pAKT downregulation in a dose- and time-dependent manner in all sensitive TNBC cells and in this way mediated the apoptotic effect of bortezomib. CIP2A governs tamoxifen-induced apoptosis in ER-negative breast cancer cells [113]. When tested for the efficacy of tamoxifen (in a panel of ER-negative breast cancer cells), tamoxifen differentially effected apoptosis in human ER-negative breast cancer cell lines as compared to ER-positive lines. Tamoxifen inhibited CIP2A in a dose-dependent manner in all apoptosis-sensitive ER-negative breast cancer cells (MDA-MB468, MDA-MB453, MDA-MB231), but not in resistant cells (HCC1937). Tamoxifen treatment downregulated CIP2A in MDA-MB468 xenograft tumors, but not in HCC1937 tumors.

Wang et al., investigated the role of CIP2A in mediating the synergism between temsirolimus (mTOR inhibitor) and cetuximab (EGFR inhibitor) in colon cancer and showed that temsirolimus mediated enhancement of the efficacy of cetuximab in colon cancer is CIP2A-dependent [114]. The mTOR protein immunoprecipitated along with CIP2A protein. Temsirolimus decreased pERK and phosphorylated v-AKT murine thymoma viral oncogene (pAKT) and decreased the interaction of CIP2A and mTOR in cell lines without the K-RAS codon 12 mutation. Temsirolimus decreased the resistance of cells to cetuximab by both inhibiting transcription of CIP2A and increasing degradation of CIP2A through the lysosomal-autophagy pathway. CIP2A was found to be a prognostic marker in colon cancer patients with weak expression of pERK or pAKT and potential biomarkers for CIP2A inhibitors include pERK and pAKT.

An increase in CIP2A expression was associated with doxorubicin resistance in breast cancer cells [55]. Since CIP2A increases the proliferation of several cancer cells, they measured the effect of CIP2A on the doxorubicin-mediated inhibition of cell proliferation. The authors' work revealed the mechanism of CIP2A regulation by doxorubicin and CIP2A-mediated doxorubicin resistance. MDA-MB231 cells showed an increase in CIP2A expression after treatment with doxorubicin, while MCF7 cells showed a decrease in CIP2A expression. The overexpression of CIP2A in MCF7 cells overcame the inhibition of cell proliferation in response to doxorubicin treatment. CIP2A expression was not affected by wild-type or mutant p53 (lack of p53 leads to doxorubicin resistance). As a regulatory mechanism of doxorubicin-mediated CIP2A expression, it was showed that phosphorylated AKT was involved in the suppression of CIP2A expression. Mutant p53 blocked doxorubicin-mediated CIP2A downregulation in HCT116 cells [55].

### Future Perspectives

As studies are revealing the exquisitely complex relationship between different oncogenic pathways and cellular functions specific to different cancer types [115-119], understanding how genetic changes upregulate the growth promoting signaling pathways in cancer cells will be the most rationale way in which researchers can develop more effective therapeutic interventions in future. Being a pleiotropic disease, cancer cells have a characteristic way of changing over time and even within a specific tumor. Cells may have different mutations and dependencies on different signaling pathways for survival or for metastatic potential [120]. New tools and technologies for genomic- or systems-level analysis, in addition to the conventional biochemistry and cell biology approaches are starting to reveal how signaling pathways of CIP2A's “oncogenic nexus” contribute to cancer development, cancer evolution, and drug resistance.

Surprisingly since CIP2A depletion did not induce any overt short-term effects on cellular morphology or viability, it is unlikely to show up in a genomic siRNA screen designed to detect dramatic phenotypic readouts due to depletion of a single protein [27]. Also CIP2A is not sufficient alone to induce tumorigenic conversion of immortalized mouse fibroblasts [2]. This characteristic of CIP2A strongly indicates that CIP2A action is mediated via its “oncogenic nexus” with other critical onco-proteins, such as RAS and MYC as well as tumor suppressor, PP2A. CIP2A antagonizes PP2A action and cooperates with RAS and MYC in regulating malignant growth and transformation.

One interesting aspect of CIP2A's “oncogenic nexus” appears to be its physiological role versus its contribution to oncogenic transformation. It is clear from the literature that CIP2A is hardly expressed in non-transformed adult tissues (except testis). Peng et al., reported that examination of the dynamic expression of p90/CIP2A during mouse development shows that p90/CIP2A protein is mainly expressed during embryo development, and becomes silent after birth [95]. The lack of physiological function of CIP2A in an adult organism raises the question regarding its role outside being an inhibitor of the tumor suppressor PP2A. Results from a more recent study by Ventela et al., revealed first physiological function for oncoprotein CIP2A [121]. They generated a CIP2A hypomorphic CIP2A^HOZ^ mouse using gene-trap technology. CIP2A^HOZ^ mice were viable and presented a normal lifespan and did not show any obvious anatomical malformations. CIP2A expression correlated with expression of spermatogonial progenitor cell self-renewal marker PLZF and testicular germ cell proliferation in mice. In human testicular spermatogonia, CIP2A and PLZF expression were shown also to correlate with Ki-67 expression. This study also demonstrated the clinical relevance regarding targeting of oncogenic CIP2A for future cancer therapies based on the fact that CIP2A expression can be systematically inhibited without severe consequences to normal mouse development and viability. The fact that (1) there is a limited role of CIP2A in adult cells and (2) CIP2A is overexpressed differentially in the tumor compartment in contrast to the adjacent non-tumor regions provides an unique opportunity to target CIP2A for therapy. Targeting CIP2A will be more specific to the tumor cells and also will have limited overall toxicity.

As a cancer target CIP2A is unique. The detailed molecular structure of CIP2A remains to be resolved. Although this poses a problem for CIP2A to be recognized as a “druggable” candidate, the uniqueness of CIP2A lies in its functions. CIP2A as an integral protein does not have any enzymatic activity. Yet it has a strong and bi-directional functional control over the enzymatic action of PP2A and oncogenic transcription factor MYC in transformed cells. CIP2A is a rare inhibitor of tumor suppressor PP2A, protein phosphatase which dephosphorylates several well-known oncogenes including MYC, beta-catenin, AKT and BCL2 [3, 4]. Our current knowledge regarding the structure, function and regulation of CIP2A with particular emphasis on its “oncogenic nexus” opens up a possibility that CIP2A can be targeted in cancers.

## SUPPLEMENTARY MATERIAL AND TABLES




