# Supplementary material for: Oncogenic nexus of cancerous inhibitor of protein phosphatase 2A (CIP2A): An oncoprotein with many hands
Source: Oncotarget. 2014 Jun 22;5(13):4581–602. doi: 10.18632/oncotarget.2127 (PMC4148086; doi:10.18632/oncotarget.2127)
Supplement: Supplementary file 2 [file oncotarget-05-4581-s002.pdf]

Supplementary Table: Reports (PMID) on role of CIP2A in different organ type cancers

Organ Type Cancers	Tumor Cell Functions	Clinical Remarks	PMID
AML	The high expression of CIP2A in HL60 cells may be related to active cell proliferation and arrest of cell differentiation.	CIP2A is over-expressed in patients with newly diagnosed/relapsed AML and the expression of CIP2A could have potential use as a clinical marker for AML relapse after treatment. CIP2A has a role in myeloid leukemogenesis.	21219591
Astrocytoma	CIP2A depletion in the astrocytoma cell lines inhibited cell growth, reduced anchorage-independent cell growth and increased apoptosis. CIP2A depletion increased caspase-3 cleavage and downregulated c-Myc, Bcl-2 and phospho-Akt expression.	CIP2A as a clinically relevant oncoprotein	23467938
B-cell Lymphoma		Increased expression of CIP2A in aggressive subtypes of B-cell lymphoma	23802697
Bladder Cancer	CIP2A is preferentially expressed in high-grade and high-stage TCC tumors, which are high-risk and invasive tumors. The study supports the role of CIP2A in bladder cancer progression.	CIP2A protein expression reported in high-grade, high-stage bladder cancer; identified the cancerous inhibitor of PP2A (CIP2A) protein as a novel bladder cancer biomarker	23342256
Bladder Urothelial cell carcinoma	Knockdown of the CIP2A expression reduced cell proliferation, anchorage-independent growth, migration,	CIP2A is an independent predictor of poor prognosis of bladder UCC patients	23275123

Supplementary Table: Reports (PMID) on role of CIP2A in different organ type cancers

	invasion, and tumor growth in xenograft model mice		
Breast Cancer	CIP2A protein expression	Potential biomarker for neo-adjuvant chemotherapeutic sensitivity and prognosis of breast cancer	23471718
Breast Cancer	Promotes the malignant growth of breast cancer cells	Associated with clinical aggressiveness of the disease and a considered as clinically relevant human onco-protein	19671842
Breast Cancer	p90/CIP2A can induce a relatively higher frequency of autoantibody response in breast cancer compared to the sera of normal individuals. The frequency of p90/CIP2A expression in breast cancer tissues was significantly higher than that in adjacent normal tissues.	Auto-antibodies against p90/CIP2A may be a useful serum biomarker for early stage breast cancer screening and diagnosis	24399648
Breast Cancer	CIP2A signature reveals the MYC dependency of CIP2A-regulated phenotypes	Results validate CIP2A's role in regulating MYC-mediated gene expression and provide a plausible novel explanation for the high MYC activity in basal-like and HER2+ breast cancers	22249265
Breast Cancer	Overexpression of CIP2A in MCF-7 cells overcame the inhibition of cell proliferation in response to doxorubicin treatment. CIP2A expression was not affected by wild-type or mutant p53. However, mutant p53 blocked doxorubicin-mediated CIP2A down-regulation	Increase in CIP2A expression is associated with doxorubicin resistance	21241697

Supplementary Table: Reports (PMID) on role of CIP2A in different organ type cancers

	in HCT116		
Breast Cancer; TN	CIP2A mediated the apoptotic effect of bortezomib		22537901
Cervical and Endometrial Carcinoma	The binding of Ets1 and Elk1 together to the proximal CIP2A promoter is absolutely required for CIP2A expression in cervical, endometrial and liver carcinoma cell lines		23117818
Cervical Cancer		Elevated CIP2A mRNA levels in cervical tissues had a sensitivity of 80% and specificity of 91% and CIP2A protein expression detection had a sensitivity of 83% and specificity of 100%, similar to that of p16INK4a, with no correlation of CIP2A expression with HPV infection, age, race, or other patient characteristics	22072119

Supplementary Table: Reports (PMID) on role of CIP2A in different organ type cancers

Cholangiocarcinoma		Increased expression of CIP2A in cholangiocarcinoma and correlation with poor prognosis. COX regression analysis implied that expression of CIP2A protein was an independent prognostic factor for cholangiocarcinoma patients	24046827
Chronic Myeloid Leukemia	The potential mechanism for disease progression is via altered phosphorylation of the oncogene c-Myc. Knockdown of CIP2A results in increased PP2A activity, decreased c-Myc levels, and a decrease in BCR-ABL1 tyrosine kinase activity	CIP2A at diagnosis of chronic myeloid leukemia is a critical determinant of disease progression	21490338
Colon Cancer	Depletion of CIP2A substantially inhibited growth of colon cell lines and reduced c-Myc levels without affecting expression or function of the upstream regulatory kinase, Akt. Expression of CIP2A was found to be dependent on MAPK activity, linking elevated c-Myc expression to deregulated signal transduction in colon cancer	CIP2A serves as an independent prognostic marker for disease-free and overall survival	24098375
Colon Cancer	The knockdown of CIP2A reduced proliferation and anchorage-independent colony formation and increased 5-fluorouracil, oxaliplatin, and SN38 efficacy in colon cancer cell lines.	CIP2A is a prognostic factor in colon cancer	22328001
Colorectal	PP2A activity is commonly	Identified	24448818

Supplementary Table: Reports (PMID) on role of CIP2A in different organ type cancers

Cancer	decreased in CRC cells	overexpression of the endogenous PP2A inhibitors SET and CIP2A and downregulation of regulatory PP2A such as PPP2R2A and PPP2R5E as contributing mechanisms to PP2A inhibition in CRC.	
Colorectal Cancer	CIP2A overexpression is associated with c-Myc expression	CIP2A shows no association with patient prognosis in colorectal cancer, but is associated with nuclear c-Myc	22310977
Cutaneous Malignant Melanoma	CIP2A immune-staining level was correlated with Breslow thickness, Clark's Level and lympho-vascular invasion. High-CIP2A expression implied poor survival for patients. Downregulation of CIP2A attenuated metastasis of CMM cells	CIP2A may serve as a novel marker to predict the prognosis for cutaneous malignant melanoma patients	24369732
Esophageal Adenocarcino ma	The expression of CIP2A and c-MYC was associated with each other, and their overexpression was found in most cases of esophageal adenocarcinoma	CIP2A and c-MYC had no effect on survival	23925667
Gastric Cancer	CIP2A and MYC appear to be regulated in a positive feedback loop	Prognostic role of CIP2A;CIP2A immune-positivity is a predictor of survival	19470954

Supplementary Table: Reports (PMID) on role of CIP2A in different organ type cancers

Gastric Cancer	CIP2A in tumor cells is required for sustained proliferation by preventing cell growth arrest, senescence, or differentiation and its expression is significantly discriminatory between normal and cancerous gastric tissue		18559589
Gastric Cancer	Helicobacter pylori enhances CIP2A expression and cell proliferation via JNK2/ATF2 signaling	H. pylori-induced upregulation of CIP2A contributes to the development and progression of gastric cancer	24398514
Head and Neck Squamous cell Carcinoma	Bortezomib inhibited CIP2A in pre-translational level in a dose- and time-dependent manner. Over-expression of CIP2A up-regulated p-Akt and protected HNSCC cells from bortezomib-induced apoptosis. Furthermore, xenograft model showed that bortezomib down-regulated CIP2A and p-Akt	CIP2A is demonstrated to be a new therapeutic target of bortezomib in HNSCC	22342571
Hepatocellular Carcinoma	Bortezomib Congeners Induce Apoptosis via CIP2A Inhibition		24335617
Hepatocellular Carcinoma	CIP2A mediates effects of bortezomib on phospho-Akt and apoptosis	Inhibition of CIP2A determines the effects of bortezomib on apoptosis and PP2A-dependent Akt inactivation in HCC	20729919
Hepatocellular Carcinoma		CIP2A is highly expressed in hepatocellular carcinoma and predicts poor prognosis	22847158

Supplementary Table: Reports (PMID) on role of CIP2A in different organ type cancers

Hepatocellular Carcinoma	CIP2A mediates bortezomib-induced autophagy	Bortezomib induces autophagy in HCC through a CIP2A-PP2A-Akt-4EBP1 pathway	23383345
High-Risk Myelodysplastic Syndromes	CIP2A was mainly expressed by the MPO-positive myeloid series of cells and partly by the CD34-positive cells in association with the expression of phosphorylated c-MYC (p-c-MYC) protein and the cell cycle-related proteins Ki-67, MCM2, and geminin	Up-regulated expression of CIP2A might play a role in the proliferation of blasts in the MDS bone marrow and in disease progression	24163288
Lung Cancer	CIP2A overexpression was associated with cigarette smoking. Silencing CIP2A by siRNA inhibited the proliferation and clonogenic activity of lung cancer cells	CIP2A could be an effective target for lung cancer drug development	21655278
Metaplasia-Dysplasia-Adenocarcinoma Sequence in the Esophagus		c-Myc overexpression is strongly associated with metaplasia-dysplasia-adenocarcinoma sequence in the esophagus	17509117
NCI-60 cell lines	Underexpression of tumor suppressor miR-375 could lead to uncontrolled CIP2A expression and extended stability of MYC		23552692
Neuroblastoma		In neuroblastoma, CIP2A was identified as an NMYC-independent prognostic factor. A statistically significant correlation was observed between the mRNA expression of Chk1 and that of CIP2A	24072747

		in neuroblastomas. Importantly,Kaplan–Meier analysis for both Chk1 and CIP2A demonstrated a significant decrease in overall and relapse-free survival in neuroblastoma patients with high expression of either of these genes.	
NSCLC	CIP2A with survivin protein expressions in human non-small-cell lung cancer correlates with prognosis	Biomarker of biological malignancy	21874565
NSCLC	Depleting CIP2A expression inhibited growth and clonogenic potential in lung cancer cell lines.	CIP2A is overexpressed in non-small cell lung cancer and correlates with poor prognosis	20842459
NSCLC	Cancerous inhibitor of PP2A is targeted by natural compound celastrol for degradation in non-small-cell lung cancer	Celastrol potentiates cisplatin's efficacy by suppressing the CIP2A-Akt pathway	24293411
Oral Cancers	Tumor suppressor miR-375 regulates MYC expression via repression of CIP2A coding sequence through multiple miRNA-mRNA interactions	Underexpression of tumor suppressor miR-375 could lead to uncontrolled CIP2A expression and extended stability of MYC, which contributes to promoting cancerous phenotypes	23552692
Oral Carcinoma and Dysplasia		CIP2A may play a significant role in oral malignant transformation	21068540
Oral Squamous cell carcinoma	CIP2A is participating in cellular transformation in oral squamous cell carcinoma		20729627
Osteosarcoma	Regulates cell proliferation and	Data identified CIP2A	24014087

Supplementary Table: Reports (PMID) on role of CIP2A in different organ type cancers

	invasion	as a critical oncoprotein	
Ovarian Cancer	Regulates cell proliferation and apoptosis;CIP2A depletion in ovarian cancer cell lines inhibited proliferation, blocked cell cycle progression, and increased paclitaxel-induced apoptosis. Furthermore, CIP2A depletion downregulated cyclin D1, c-myc, phospho-Rb, Bcl-2, and phospho-AKT expression.	Role of CIP2A as a clinically relevant oncoprotein and establish CIP2A as a promising therapeutic target of ovarian cancer	22923389
Pancreatic Ductal Adeno-carcinoma	Epithelial-mesenchymal transition markers	Predictive of poor prognosis	23568706
Prostate Cancer	Expression of CIP2A was increased in prostate cancer epithelium as compared with the benign hyperplastic epithelium. The expression of CIP2A was associated with high Gleason scores and among patients treated with radical prostatectomy	CIP2A protein is increased in prostate cancer specimens and its expression is associated with poorly differentiated and high-risk tumors.	20964854
Renal Cell Carcinomas	Cancerous inhibitor of protein phosphatase 2A depletion by siRNA down-regulated c-Myc expression and attenuated the migration and invasion of RCC cells.	Expression of CIP2A correlates with tumor invasion, metastasis and patients' survival	22075943
Serous Ovarian Cancer	Cancerous inhibitor of protein phosphatase 2A overexpression was also associated with EGFR protein expression (P=0.006) and EGFR amplification (P=0.043). Strong cytoplasmic CIP2A immune-positivity predicted poor outcome	Prognostic role of CIP2A; CIP2A associates with reduced survival and parameters associated with high grade in ovarian cancer patients, and may thus be one of the factors that	21897396

Supplementary Table: Reports (PMID) on role of CIP2A in different organ type cancers

	in ovarian cancer patients	identify aggressive subtype (type II) of this disease	
Tongue Cancer	High CIP2A expression characterizes aggressive disease	High CIP2A immuno-reactivity is an independent prognostic indicator in early-stage	21610708
